# Undetectable high-performance liquid chromatography haemoglobin A1c on variant haemoglobin E phenotype: a case report

**DOI:** 10.11613/BM.2023.030801

**Published:** 2023-10-15

**Authors:** Nadia Sadriani, Ferdy Royland Marpaung

**Affiliations:** 1 Clinical Pathology Specialist Programme, Department of Medicine, Dr Soetomo Academic Hospital/Faculty of Medicine Universitas Airlangga, Surabaya, Indonesia; 2 Department of Clinical Pathology, Dr Soetomo Academic Hospital/Faculty of Medicine, Universitas Airlangga, Surabaya, Indonesia

**Keywords:** diabetes mellitus type 2, HbA1c, high-performance liquid chromatography, haemoglobin E, glycated albumin

## Abstract

The gold standard for long-term monitoring of diabetic patients is glycated haemoglobin (HbA1c), which is routinely tested for glycaemic control. Furthermore, the National glycohemoglobin standardization program (NGSP) has designated high-performance liquid chromatography (HPLC) as the reference method for HbA1c measurement. A woman from the Sumba tribe, Indonesia, aged 52, visited the Internal Medicine Clinic for a routine check-up. She had been taking diabetic and hypertension medicines on a regular basis for over 10 years. The HPLC procedure yielded “no result” for the patient’s HbA1c assessment and there was no peak on the HPLC graphic. However, there was a discrepancy between the data history of HbA1c measured by turbidimetric method (average of 51 mmol/mol, reference range < 48 mmol/mol), fasting blood glucose (average of 7.7 mmol/L, reference range < 7.0 mmol/L) and 2-hour plasma glucose (average of 13 mmol/L, reference range < 11.1 mmol/L). Glycated albumin was 3.1 mmol/L (reference range 1.8-2.4 mmol/L). Haemoglobin electrophoresis identified homozygote haemoglobinopathy E (HbE). Patients with haemoglobin variants are proposed to utilize glycated albumin.

## Introduction

Globally, the number of people with diabetes mellitus (DM) in adulthood reaches more than 537 million patients. Interestingly, three quarters of those come from low and middle-income countries, with half of the population undiagnosed, resulting in a very high death rate (over 6 million). Data also shows that in Indonesia, there are nearly 20 million cases of DM, making Indonesia the fifth-largest DM population in the world (*1*). According to the American diabetes association (ADA), in addition to fasting blood glucose (FBG) and 2 hour-plasma glucose (2h-PG), the glycated haemoglobin A1c (HbA1c) ≥ 48 mmol/mol is used to diagnose DM (*2*). However, the HbA1c should be measured in a laboratory using a method that is certified by National glycohemoglobin standardization program (NGSP) and standardized to the Diabetes control and complication trial (DCCT) assay. The gold standard for extended evaluation in diabetes patients, HbA1c, is also the regular test for glycemic control monitoring. In laboratories, the high-performance liquid chromatography (HPLC) is the most commonly used method for measuring HbA1c, followed by immunoassay, boronate affinity HPLC, ion-exchange HPLC, enzymatic assays and latex agglutination (*3*).

Here we present a case of a 52-years-old woman with diabetes who had history of high FBG and 2h-PG but had no HbA1c peak detected by HPLC method. The haemoglobin (Hb) electrophoresis showed the presence of haemoglobin E (HbE). The aim of this case report is to demonstrate the additional benefit of using the HPLC method in the management of DM patients with HbE.

## Case presentation

The informed consent was obtained from the subject. This study was approved by the Dr. Soetomo General Regional Hospital, Surabaya, Indonesia Research Ethics Committee (Date: December, 15^th^ 2022, Decision No: 1163/LOE/301.4.2/XII/2022).

**Figure 1:**
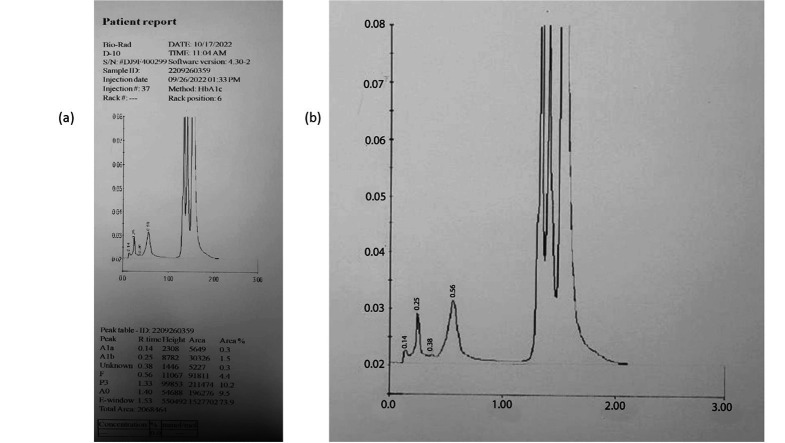
HPLC graph shows no peak and no result in HbA1c with (a) full image and (b) detailed view. HPLC - high-performance liquid chromatography. HbA1c – glycated haemoglobin A1.

**Figure 2:**
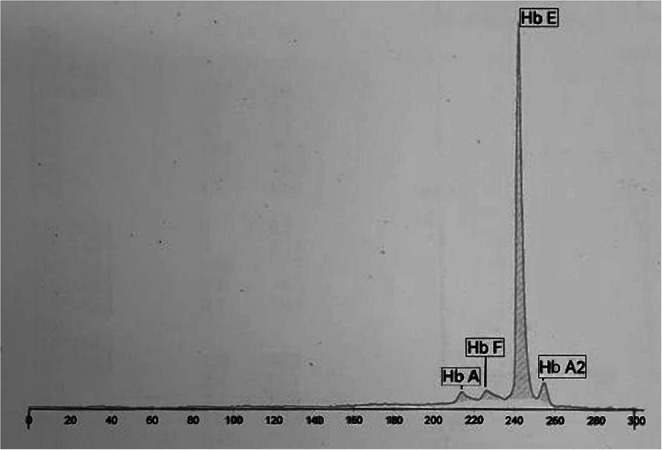
Haemoglobin (Hb) electrophoresis. Results: HbA 2.9% (reference range 96.8-97.8%), HbF 3.8% (reference range ≤ 0.5%), HbE 89.4%, HbA_2_ 3.9% (reference range 2.2-3.2%).

**Figure 3:**
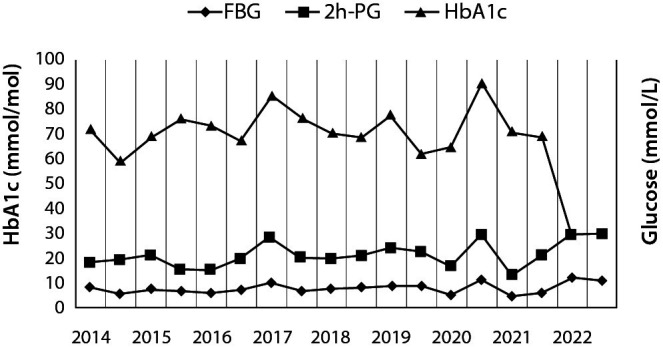
History of FBG and 2h-PG and HbA1c (immunoturbidimetric method). FBG - fasting blood glucose. 2h-PG - 2 hour-plasma glucose (2h-PG). HbA1c – glycated haemoglobin A1.

A 52-year-old woman, Sumba tribe, Indonesia, visited the Internal Medicine Clinic for a routine six-month check-up. She had suffered from diabetes and hypertension for almost ten years but was regularly checked with the doctor and got the oral medication for DM. There was no complaint from the patient at that time. The patient was generally well conscious, with blood pressure of 140/95 mmHg, a pulse of 88 *per* minute, a respiration rate of 18 times *per* minute, and an axilla temperature of 36.6 °C. Other physical examinations were within normal limits. Laboratory testing results are shown in Table 1 and Figures 1-3. The Mentzer Index (mean cell volume/red blood cell count) was < 13, indicating the possibility of thalassemia, haemoglobinopathy, and iron deficiency anaemia. A peripheral blood smear showed hypochromic microcytic anisopoikilocytosis (normocytic, many ovalocytes, and target cells). Therefore, the clinical pathologist performed laboratory testing of serum iron (SI), total iron-binding capacity (TIBC), and haemoglobin electrophoresis. The clinicians did not report the genetic trait, since there were no complaints related to the genetic trait and the haematological result was relatively normal.

**Table 1:** Laboratory results

**Parameter (unit)**	**Result**	**Reference range/cut-off**
**Haematology results**		
Haemoglobin (g/L)	113	110-147
MCV (fL)	60.6	86.7-102.3
MCH (pg)	20.2	27.1-32.4
RBC (x10^12^/L)	5.74	3.69-5.46
**Biochemistry results**		
FBG (mmol/L)	10.8	< 7.0
2h-PG (mmol/L)	18.4	< 11.1
HbA1c (mmol/mol)	No result	< 48
Glycated albumin (mmol/L)	3.1	1.8-2.4
Fasting insulin (mIU/L)	10.61	< 25
C-peptide (nmol/L)	2.72	0.17-0.83
BUN (mmol/L)	5.6	3.6-7.1
CREA (µmol/L)	82.2	44.2-106.0
Fe (µmol/L)	5.04	3.09-13.26
TIBC (µmol/L)	23.17	22.11-39.79
eGFR (mL/min/173 m^2^)	71	≥ 90
LD (U/L)	169	100-190
TBIL (µmol/L)	11.3	3.42-17.1
DBIL (µmol/L)	2.9	0.00-3.24
**Urinalysis results**		
SG	1.004	1.000-1.030
pH	5.5	4.5-6.0
Leukocyte	+ 3	Negative
Nitrite	Negative	Negative
Protein	+ 2	Negative
Glucose	Trace	Negative
Ketones	Negative	Negative
Urobilinogen	Normal	< 0.1
Bilirubin	Negative	Negative
Colour	Yellow	Yellow
Clarity	Turbid	Clear
Erythrocyte (blood)	+ 2	Negative
ACR (A:C) (mg/gCr)	≥ 300	< 30 mg/gCr
PCR (P:C) (g/gCr)	≥ 50	< 0.15 g/gCr
MCV - mean corpuscular volume. MCH - mean corpuscular haemoglobin. RBC - red blood cell. FBG - fasting blood glucose. 2h-PG - 2-hour plasma glucose. BUN - blood urea nitrogen. CREA - creatinine. Fe - iron. TIBC - total iron binding capacity. LD - lactate dehydrogenase. eGFR - estimated glomerular filtration rate. TBIL - total bilirubin. DBIL - direct bilirubin. SG - specific gravity. ACR - albumin creatinine ratio. PCR - protein creatinine ratio.		

The results of the patient’s HbA1c examination, performed by HPLC method (D-10, Biorad, Hercules, USA), showed “no result” and there was no peak shown in the graphic (Figure 1). The D-10 haemoglobin testing system is based on the chromatographic separation of the analytes by ion-exchange HPLC (*4*, *5*). The samples are automatically diluted on the D-10 and injected into the analytical cartridge, which then delivers a programmed buffer gradient of increasing ionic strength to the cartridge when the haemoglobin is separated based on their ionic interactions with the cartridge material. The separated haemoglobin then passes through the flow cell of the filter photometer, where changes in the absorbance at 415 nm are measured (*4*). The method’s coefficient of variation (CV) was 1.2% with a cut-off < 46 mmol/mol for non-diabetic concentrations (*5*).

Three months prior, the HbA1c method was changed from immunoturbidimetric (Dimension EXL2, Siemens, Erlangen Germany) to HPLC (D-10, Biorad, Hercules, USA). Because the HPLC gave no results, the clinician requested an effort to see the over-time observation of glycemic index. In addition, the clinician also drew attention on previous HbA1c results (immunoturbidimetric), which did not correspond to the patient’s blood glucose (Figure 3). Average results for FBG (7.7 mmol/L) and 2h-PG (13 mmol/L) were relatively high; however average HbA1c (immunoturbidimetric) was relatively normal (51 mmol/mol), implying that the latter should have a higher HbA1c. We suggested performing an Hb electrophoresis, because the patient was suspected to have thalassemia or haemoglobinopathy. The following results were obtained by Hb electrophoresis: HbA 2.9%, HbF 3.8%, HbE 89.4%, and HbA2 3.9%. As a result, it was concluded that homozygote HbE exists (Figure 2). The working diagnosis was DM type 2, hypertension, homozygote HbE and renal complications.

The clinician advised the patient to exercise, changed the oral DM medication to insulin and referred the patient for control to the haematologic-oncologic division due to HbE. The haematologic-oncologic division specialist provided no particular therapy, merely an advice to frequently examine the haemoglobin concentration every three months. Another recommendation for this patient was to examine routine laboratory parameters, including blood glucose, FBG, and 2h-PG (every month), monitor complications of diabetic kidney disease, including glycated albumin (GA) and/or fructosamine concentration every month, and albumin to creatinine (A/C) ratio or protein to creatinine (P/C) ratio every 6 months.

## Discussion

Indonesia has a population of more than 260 million people, with 5% of the population possibly carrying the thalassemia gene. Several regions in Indonesia have different genetic patterns and heterogeneity in HbE cases. For example, in Sumba (Sumba Island), HbE cases are relatively high, namely 33%. Lack of public awareness of treating asymptomatic disease in Indonesia may be the reason for the high number of beta-thalassemia/HbE in underreported cases (*6*). Haemoglobin E carriers generally do not have any medical signs and symptoms, but in combination with thalassemia (HbE/β-thalassemia), they may also develop mild to severe persistent anaemia (*7*). This patient was from the Sumba tribe, which corresponds to predominance of HbE. However, there were no specific complaints that led to HbE/thalassemia diagnosis in this patient, only frequent feeling of weakness and dizziness, ignored by the patient.

The patient had a history of uncontrolled FBG and 2h-PG. Still, HbA1c was always in the “normal range” when measured using the immunoturbidimetric method. However, when determined by the HPLC method, the HbA1c was undetectable. This may be caused (according to the package insert):

- low sample volume < 2 mL;
- presence of peak variant window >60%, when it is recommended to dilute the sample to 1:300. If the result is still “no result”, then potential laboratory diagnosis is beta-thalassemia phenotype with Hb variant homozygote. The confirmation will require haemoglobin analysis;
- if no peak appears in all samples in 1 batch run, there may be a D10 hardware problem.

In our case, there was sufficient sample volume and the sample has been diluted 1:300, but still there was no result (*4*, *5*). Also, no peak results only appeared in this patient out of 40 samples. We concluded that this result was caused by high HbE (89%) that detected HbE homozygote.

Some methods to examine HbA1c include immunoassay, boronate affinity HPLC, ion-exchange HPLC, and enzymatic assays, latex agglutination (*8*, *9*). The HPLC method is very popular because it has one advantage: the ability to remove labile components, successively pre A1c or Schiff base. This component derived from the first phase of glycation of haemoglobin HbA, which is a rapid, reversible and dependent phase of the plasma glucose concentration (*10*). A falsely low HbA1c concentration can result from several conditions, including prolonged stay at high altitude, pregnancy, bleeding, blood transfusion, erythropoietin management, iron supplementation, haemolytic anaemia, chronic kidney failure, liver cirrhosis, alcoholism, sickle cell anaemia and spherocytosis (*3*, *8*, *10*-*15*).

The enzymatic and immunoassay measure cannot give any information about Hb variants, which should be considered when utilizing this strategy for HbA1c alone (*16*). Boronate affinity HPLC examination is interfered by rare Hb variants; thus, unable to detect Hb variants. The recommended procedure is ion-exchange HPLC compared to capillary electrophoresis, which is susceptible to interferences from Hb variants, Hb derivatives and HbF. For more than 8 years, our hospital has used solely the immunoturbidimetric method for HbA1c, which cannot distinguish between normal and Hb disease patients HbA1c levels. When we switched to HPLC, we discovered that the presence of Hb variations interfered with the HPLC process. Ideally, we can use the HPLC approach to screen for Hb variations while doing HbA1c for the first time.

Whenever there are unexpected Hb fluctuations, the HbA1c implications should be evaluated in the patient’s clinical setting. When inconsistencies are observed between a patient’s blood glucose and laboratory-measured HbA1c, a falsely elevated or decreased HbA1c result must be considered. Any result that does not correlate with the clinical situation should also be investigated (*17*, *18*). If an abnormal HbA1c concentration or chromatogram is detected, then further suggestion of haemoglobin examinations as Hb electrophoresis, should be communicated with the clinician. It is recommended to perform haemoglobin electrophoresis and genetic testing to exclude haemoglobinopathy. The accuracy of HbA1c measurement in patients with haemoglobinopathy depends on the method used. Gao *et al.* have shown that this condition does not affect immunoassay and enzymatic methods. However, any decrease in erythrocyte life span in cases of haemoglobinopathy with anaemia may affect the result (*13*).

Because it is unaffected by other haemoglobin and erythrocyte disorders and can precisely reflect glycaemic control and provide a glycaemic state of past two weeks, GA test is superior the glycated haemoglobin test. Glycated albumin has a strong correlation with HbA1c and can serve as a substitute for the glycaemic index in diabetic nephropathy patients (*19*). However, GA shows anomalous values in patients with abnormal albumin metabolism (*19*, *20*).

If different methods for measuring HbA1c concentrations are not available in the laboratory, GA, oral glucose tolerance test (OGTT), blood glucose self-monitoring, and dynamic blood glucose monitoring are also options. Glycated albumin is the second indicator to evaluate the extent of average blood glucose and is not affected *via* abnormal haemoglobin (*11*). In regions with a high prevalence of haemoglobinopathy, GA’s position must be emphasized in blood glucose control assessment and diabetes screening (*21*). Screening based totally on affected person’s HbA1c concentration and fasting blood glucose, might lead to failure in diagnosing pre-diabetes (*13*).

In the case of undetectable HbA1c by HPLC due to the presence of HbE, we should be cautious, especially in an area with a high incidence of HbE. The ability to detect and diagnose the presence of haemoglobin variations is an advantage of the HPLC method. Additional tests, such as haemoglobin electrophoresis, may be performed.
